# Association between polymorphisms rs2228001 and rs2228000 in *XPC* and genetic susceptibility to preeclampsia: a case control study

**DOI:** 10.1186/s12884-021-04242-1

**Published:** 2021-11-22

**Authors:** Jingli Wang, Chengcheng Guan, Jing Sui, Yucui Zang, Yuwen Wu, Ru Zhang, Xiaoying Qi, Shunfu Piao

**Affiliations:** 1 Medical Genetic Department, The Affiliated Hospital of Qingdao University, Qingdao, Shandong China; 2 The Prenatal Diagnosis Center, The Affiliated Hospital of Qingdao University, Qingdao, Shandong China; 3 Obstetrical Department, The Affiliated Hospital of Qingdao University, Qingdao, China; 4 Department of Biochemistry and Molecular Biology, Qingdao University Medical College, Qingdao, 266003 China

**Keywords:** Chinese Han, Shandong province, DNA repair, *XPC*, Polymorphism, Preeclampsia

## Abstract

**Background:**

Xeroderma pigmentosum complementation group C (XPC) is a DNA damage recognition protein that plays an important role in nucleotide excision repair and can reduce oxidative stress, which may be involved in the development of preeclampsia (PE). Therefore, the aim of this study was to explore whether *XPC* polymorphisms were relevant to the genetic susceptibility to PE in Chinese Han women.

**Method:**

A total of 1276 healthy pregnant women were included as the control group and 958 pregnant women with PE as the case group. DNA was extracted from peripheral blood samples to perform genotyping of loci rs2228001 and rs2228000 in *XPC* through real-time quantitative polymerase chain reaction (PCR). The relationship between *XPC* and susceptibility to PE was evaluated by comparing the genotypic and allelic frequencies between the two groups of pregnant women.

**Results:**

Polymorphism of rs2228000 may be associated with PE risk and allele T may play a protective role (genotype, χ2 = 38.961, *P* < 0.001 and allele χ2 = 21.746 *P* < 0.001, odds ratio (OR) = 0.885, 95% confidence interval (CI) = 0.840-0.932). No significant difference was found between the two groups in rs2228001,(genotype χ2 = 3.148, *P* = 0.207 and allele χ2 = 0.59, *P* = 0.442, OR = 1.017, 95% CI = 0.974–1.062). When the frequencies of genotypes and alleles for early- and late-onset PE, mild PE and severe PE were compared with those of controls, the results were consistent with the large clinical sample.

**Conclusion:**

Our data suggest that the genetic variant rs2228000 in XPC may be associated with PE risk in Chinese Han women, and that pregnant women with the TT genotype have a reduced risk of PE. Further investigations are needed to confirm these findings in other regions or larger prospective populations.

## Introduction

Preeclampsia (PE) is defined as a special disease that occurs after 20 weeks of pregnancy, with hypertension and proteinuria or one of the following characteristics, thrombocytopenia, liver and kidney function damage, and pulmonary oedema [1], and seriously affects maternal and child health and even leads to death or morbidity during the perinatal period [2]. Although the potential harm of PE has been extensively studied, its pathogenesis remains poorly understood, and many factors may be involved in the development of PE, including inflammatory reactions, placental ischemia, vascular endothelial dysfunction, genetic factors, and oxidative stress [3–5].

Compared with normotensive pregnant women, patients with PE are in a state of extremely increased oxidative stress because of the decreased antioxidant capacity. Excess production of reactive oxygen species (ROS) can lead to vascular endothelial and DNA damage, which may be involved in the pathogenesis of PE by blocking trophoblast invasion and placental formation [6, 7]. A comet assay revealed significantly increased levels of DNA damage in the placenta of PE model rats [8]. As a sensitive indicator of DNA damage, phosphorylated H2AX was highly expressed not only in PE placentas but also in maternal metaphase stromal cells cultured in vitro with oxides [9]. Consistent with this result, Takagi et al. [10] found that 8-hydroxy-20-deoxy-guanosin (8-OHdG), a representative DNA damage marker, was detected at higher concentrations in the serum of patients with PE than in healthy pregnant women. There are enzymes related to DNA repair in the human body that can repair these damaged DNA and maintain a stable state of genetic material [11]. Therefore, it is essential to explore the role of genetic polymorphisms in the DNA repair system in PE pathogenesis based on DNA damage.

**Fig. 1:**
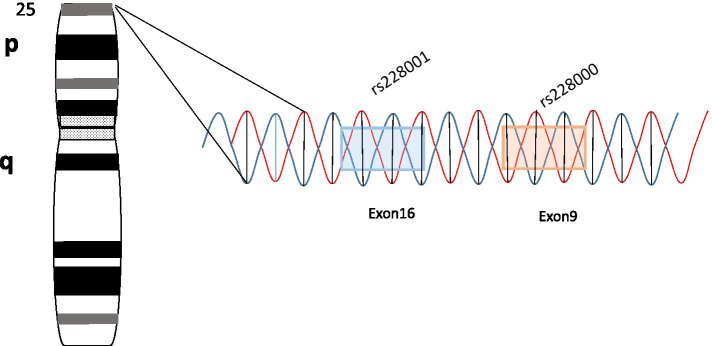
A schematic representation of rs228001 and rs228000

As a damage recognition protein of DNA, the gene Xeroderma pigmentosum complementation group C (*XPC*), which belongs to one of the key genes in the nucleotide excision repair system [12], repairs the damaged DNA by nucleotide excision to reduce the damage to DNA, and then reduces the attack of oxidative stress on organisms [13]. The XPC proteins have specific roles in cell protection and repair and tolerance of ROS-induced DNA damage [14]. In addition, reducing or silencing the expression of *XPC* increases the level of intracellular ROS, which increases oxidative stress and ultimately leads to oxidative DNA damage [15]. Two nonsynonymous single nucleotide polymorphisms (SNPs), c.2815G > T (p.Q939E, rs2228001) in exon 16 and c.1496C > T (p.A499V, rs2228000) in exon 9 of *XPC* may alter the capacity of XPC and modulate risk of various cancers [16, 17]. The positions of the two SNPs on the chromosomes are shown in Fig. 1. Currently, there is a lack of research on the association between these two SNPs and PE susceptibility. Therefore, the present study aimed to investigate the relationship between polymorphisms of rs2228001 and rs2228000 in *XPC* and the susceptibility to PE in the Chinese Han population, which further provides an experimental basis for a follow-up clinical study on PE.

## Materials and methods

### Subjects

We enrolled 958 patients with PE and 1276 normal pregnant women at the Linyi People’s Hospital, Zaozhuang Peoples’ Hospital, Heze People’s Hospital, Yantaishan Hospital, and the Affiliated Hospital of Qingdao University from January 2019 to November 2020 as the case and control groups. This study was approved by the ethics committee of Affiliated Hospital of Qingdao University, and informed consent were obtained from all participants. The diagnostic criteria for PE were based on the ACOG Practice Bulletin No. 202: Gestational Hypertension and Preeclampsia [1]. The conditions for inclusion in the case group were the presentation of normal blood pressure before 20 weeks of gestation, whereas ≥140/90 blood pressure after 20 weeks of gestation, ≥0.3 g/24 h proteinuria, or one of following symptoms, such as headache, blurred vision, thrombocytopenia, liver and kidney function damage and pulmonary oedema. According to the guidelines, PE patients with systolic blood pressure > 160 mmHg or diastolic blood pressure > 110 mmHg are classified as the severe PE group [1]. Early-onset PE was diagnosed before 34 weeks of pregnancy, whereas late-onset PE was diagnosed after 34 weeks of gestation. The conditions of the control group included ≥26 years old; ≥30 weeks of gestation; no disease of kidney disorders; foetal macrosomia; chronic hypertension, diabetes mellitus, and cardiopathy; no abnormality in the liver, kidney, and blood coagulation; and no history of transfusion immunity, obstetric complications such as threatened abortion, premature rupture of membrane, placenta previa, assisted reproduction, and twin or multiple pregnancy.

### Genetic studies

Genomic DNA was extracted from 200 μL of peripheral venous blood using a Qiagen DNA extraction kit (Qiagen, Hilden, Germany). Genotyping for polymorphisms of rs2228000 and rs2228001 in *XPC* was performed using real-time quantitative polymerase chain reaction (PCR). The TaqMan probe was designed by American Applied Bio System Inc. The rs2228000 primers were 5′-CTCTGATCCCTCTGATGAGGATTC-3′ (forward) and 5′- CCACACCGTGCACACAGTCT-3′ (reverse), the rs2228001 primers were 5-CAGAAGCGGCCAGGATACTG-3′ (forward) and 5′-TGTTCTGTAGCTCAAAGGGTGAGT-3′ (reverse). The total volume of the PCR mixture was 25 μL consisting of 1.25 μL 20× SNP Genotyping Assay, 12.5 μL 2× PCR Master Mix, and 11.25 μL DNA and DNase-free water. PCR amplification was performed using a C1000™ cycle instrument and a CFX96™ real-time system. The cycling conditions were as follows: 3 min at 95 °C for predegeneration; a total of 45 cycles of 15 s at 95 °C and 1 min at 60 °C; and 5 min at 72 °C for extension. The genotype was evaluated using BioRad CFX Manager 3.0. We used Haploview 4.2 softwares to analyze the haplotypes of these two SNPs.

### Statistical methods

Statistical analysis of experimental data was performed using the Statistical Package for the Social Sciences version 23.0. Demographic and clinical data such as age, gestational age, pregnancy times, blood pressure, and abortion times were analysed using a *t*-test to compare differences between the controls and cases. The genotypic and allelic frequencies were compared using the chi-square test (if expected values were below 5, the Fisher’s exact test was used). The distribution of genotypic and allelic frequencies in the control group was checked using the Hardy-Weinberg equilibrium, which ensured that the data were representative of the control group. The 95% confidence intervals (CIs) and odds ratios (ORs) were used to determine the relative risk degree. Considering Bonferroni adjustments, statistical significance was set at *P* < 0.025 for chi-square test.

## Results

### Demographic and clinical characteristics

The comparison of the demographic and clinical characteristics between the PE cases and controls is presented in Table 1. No significant differences were found between the patients with PE and healthy women across maternal age, times of gravidity, and number of abortions (*P* > 0.05); however, patients with PE had an earlier admission and delivery gestational weeks, lower foetal weight, and higher blood pressure than the controls (*P* < 0.001).

**Table 1:** Demographic and clinical characteristics of the PE and control groups

Characteristics	PE (958)	Controls (1279)	t	***P***-vaule
Maternal ages	30.24 ± 3.90	30.14 ± 3.53	0.472	0.637
Times of gravidity	2.22 ± 1.28	2.23 ± 1.19	−0.161	0.872
Gestational ages	35.22 ± 3.49	39.17 ± 1.38	−34.28	< 0.01
Number of abortion	1.42 ± 0.71	1.55 ± 0.85	−2.493	< 0.001
Birth weight of offspring	2.61 ± 0.92	3.40 ± 0.38	−24.479	< 0.001
Systolic blood pressure(mmHg)	161.01 ± 18.7	114.87 ± 9.95	−72.03	< 0.001
Diastolic blood pressure(mmHg)	104.63 ± 13.73	73.51 ± 7.72	−65.52	< 0.001

### Genotypic and allelic frequencies

The genotypic distribution of controls was in Hardy–Weinberg equilibrium for both SNPs (rs2228000: χ2 = 0.024, *P* = 0.877; rs2228001: χ2 = 0.0005, *P* = 0.983). Statistical analysis indicated there was statistical difference in the genetic distributions of rs2228000 in *XPC* between the case and control groups (genotype, χ2 = 38.961, *P* < 0.001 and allele χ2 = 21.746 *P* < 0.001, odds ratio (OR) = 0.885, 95% confidence interval (CI) = 0.840-0.932). However, rs2228001 may be not related to the occurrence of PE (genotype χ2 = 3.148, *P* = 0.207 and allele χ2 = 0.59, *P* = 0.442, OR = 1.017, 95% CI = 0.974-1.062) (Table 2).

**Table 2:** Genotype and allele frequencies in PE and control groups

	Cases no.	Controls no.	χ2	***P***-value	OR	95%CI
***rs2228000***						
Genotype						
CC	189	232	38.961	< 0.001		
CT	510	541				
TT	259	503				
TT	259	503	37.342	< 0.001	0.686	0.606-0.777
CC + CT	699	773				
Alleles						
C	888	1005				
T	1028	1547	21.746	< 0.001	0.885	0.840-0.932
***rs2228001***						
Genotype						
TT	408	545	3.148	0.207		
GT	444	560				
GG	106	171				
TT	408	545	0.003	0.954	0.997	0.905-1.099
GT + GG	550	731				
Alleles						
T	1260	1650	0.59	0.442	1.017	0.974-1.062
G	656	902				

Comparing patients with mild PE with the control group, rs2228000 showed a distribution frequency difference in genotypes (for rs2228000, genotypic frequency, χ2 = 8.849, *P* = 0.012; allelic frequency, χ2 = 2.389, *P* = 0.122, OR = 0.924, 95% CI = 0.832-1.026; for rs2228001, genotype χ2 = 0.23, *P* = 0.892, and allele χ2 = 0.12, *P* = 0.729, OR = 1.016, 95% CI = 0.931-1.108). For the severe PE group and the control group, the genotype and allele frequencies of the polymorphism rs2228000 were different (for rs2228000, genotypic frequency, χ2 = 36.169, *P* < 0.001; allele frequency, χ2 = 22.214, *P* < 0.001, OR = 0.884, 95% CI = 0.827–0.926; for rs2228001, genotype χ2 = 5.012, *P* = 0.082, and allele χ2 = 0.001, *P* = 0.97, OR = 1.001, 95% CI = 0.947-1.058) (Table 3).

**Table 3:** Genotype and allele frequencies between mild or severe PE and control groups

Group	N	rs2228000					rs2228001				
		CC	CT	TT	C	T	TT	GT	GG	T	G
Mild PE	150	25	82	43	132	168	65	67	18	197	103
Control	1276	232	541	503	1005	1547	545	560	171	1650	902
χ2		8.849			2.389		0.23			0.12	
*P*-value		0.012			0.122		0.892			0.729	
OR						0.924				1.016	
95%CI				0.832-1.026					0.931 ~ 1.108		
Severe PE	808	164	428	216	756	860	343	377	88	1063	553
Control	1276	232	541	503	1005	1547	545	560	171	1650	902
χ2		36.139			22.214		3.361			0.551	
*P*-value		< 0.001			< 0.001		0.186			0.458	
OR						0.878				1.017	
95%CI				0.831-0.928					0.972 ~ 1.065		

Consistent with above results, there were significant differences in genetic distribution of rs2228000 between control group and early-onset PE group (genotype: χ2 = 13.286 *P* = 0.001; allelic: χ2 = 9.098, *P* = 0.003, OR = 0.909, 95% CI = 0.852-0.969) or lately-onset PE group (genotype: χ2 = 48.54 *P* < 0.001; allelic: χ2 = 14, *P* < 0.001, OR = 0.884, 95% CI = 0.827-0.926). No differences in the genetic distribution of rs228001 were found between the early-onset PE group and the control group (genotypic χ2 = 1.43, *P* = 0.489; allelic χ2 = 1.388, *P* = 0.239, OR = 1.033, 95% CI = 0.98–1.089). Comparing the genotypic and allelic frequencies of the late-onset and control groups of rs2228001, no differences were found in genotypic frequency (χ2 = 5.012, *P* = 0.082) and allelic frequency (χ2 = 0.001, *P* = 0.97, OR = 1.001, 95% CI = 0.947-1.058) (Table 4).

**Table 4:** Genotype and allele frequencies between early-onset or late-onset PE and control

Group	N	rs2228000					rs2228001				
		CC	CT	TT	C	T	TT	GT	GG	T	G
Early-onset PE	486	112	226	148	450	522	219	211	56	649	323
Control	1276	232	541	503	1005	1547	545	560	171	1650	902
χ2		13.286			9.098		1.43			1.388	
*P*-value		0.001			0.003		0.489			0.239	
OR						0.909				1.033	
95%CI				0.852-0.969					0.98-1.089		
Late-onset PE	472	77	284	111	438	506	189	233	50	611	333
Control	1276	232	541	503	1005	1547	545	560	171	1650	902
χ2		48.54			14		5.012			0.001	
*P*-value		< 0.001			< 0.001		0.082			0.97	
OR						0.884				1.001	
95%CI				0.827 ~ 0.926					0.947-1.058		

**Fig. 2:**
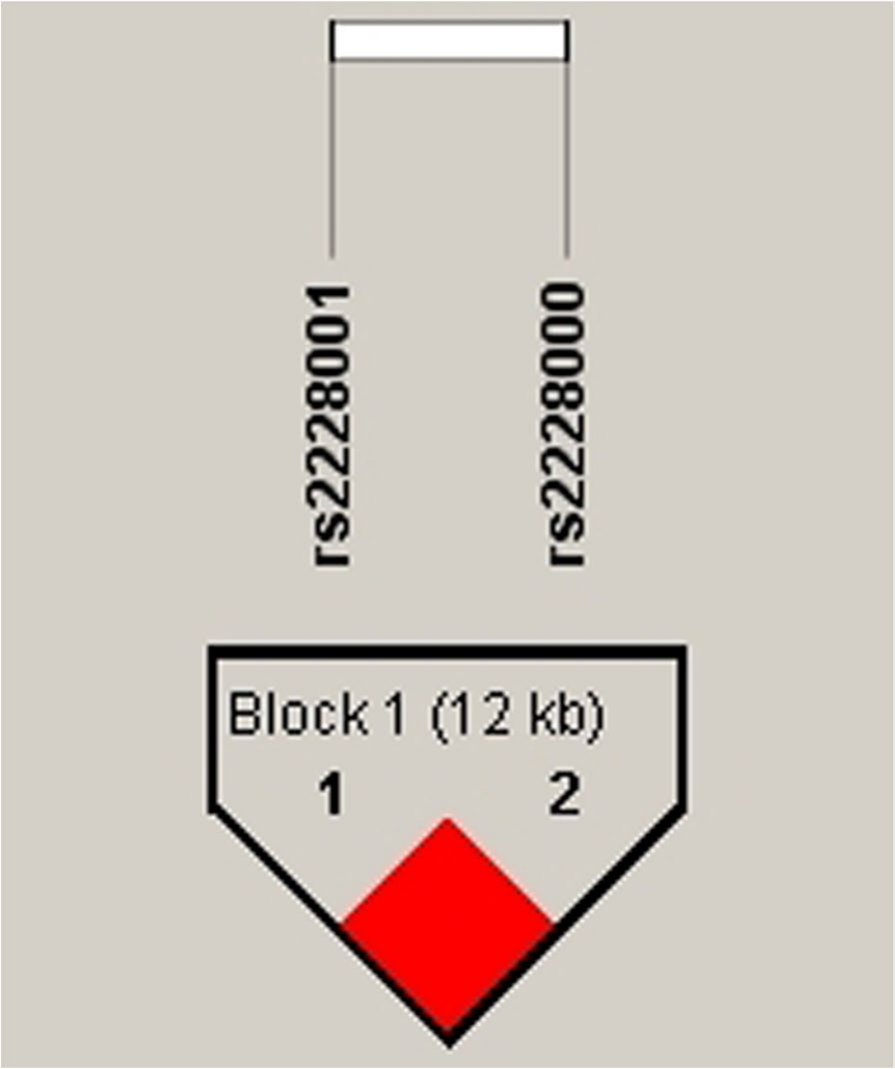
The linkage disequilibrium association between rs2228001 and rs2228000

In addition, we performed interaction and haplotype analysis of these two SNPs and found that there is an linkage disequilibrium between these two loci (Fig. 2) and that haplotypes of AC and AT may be involved in the pathogenesis of PE (Table 5) (AC: χ2 = 21.75, *P* < 0.001; AT: χ2 = 21.37, *P* < 0.001).

**Table 5:** Relationship between haplotype interaction and PE

Haplotype	Frequency	χ2	*P*-value
TT	0.424	21.75	< 0.001
GT	0.349	0.59	0.44
GC	0.228	21.37	< 0.001

## Discussion

There are many studies on the role of genetic polymorphisms in susceptibility to female diseases such as uterine fibroids, recurrent spontaneous abortion, and polycystic ovary syndrome (PCOS). The levels of anti-Müllerian hormone (AMH) were found to be significantly higher in patients with PCOS than in healthy women; however, findings regarding the association between *AMH* polymorphisms and the development of PCOS were inconsistent. Therefore, Wang et al. conducted a meta-analysis of five studies and found that AMH genetic variants may not be related to PCOS risk [18]. A genome-wide association study in a Japanese population revealed that nine SNP loci were considered risk factors for uterine leiomyoma, further indicating the important role of genetic factors in uterine leiomyoma [19]. Salimi et al. [20] suggested that SNPs of the HOX transcript antisense RNA gene, such as rs12826786, rs920778, and rs1899663, are related to the development of RSA. Previous studies have reported that women whose mothers and sisters have a history of PE have an elevated risk of PE, which provides evidence for the important role of genetic factors in PE. A common complication of pregnancy, PE is responsible for 7 to 10% of maternal morbidity worldwide [21–23], and is caused by many factors, including endothelial damage, inflammatory response, oxidative stress, and placental factors; among which the role of oxidative stress should not be ignored [5, 24]. Patients with PE are in a state of increased oxidative stress, and excessive amounts of oxidants can result in damage to lipids, proteins, and DNA. Damaged DNA can be repaired by DNA repair mechanisms, which are essential for the stability of the genetic material.

However, only a few studies have reported an association between DNA repair system polymorphisms and PE risk. A previous study has shown that in the Iranian population, Arg399Gln of X-ray repair cross-complementing group 1 (XRCC1) is associated with PE susceptibility, and the allele 399Gln is considered a risk factor for PE [25]. This result contradicted another study in the Mexican population in which xeroderma pigmentosum group D (XPD) Lys751Gln, XRCC Arg399Gln, and XRCC3 Thr241Met polymorphisms were not associated with the risk of PE [26]. This discrepancy in results may be due to racial differences. Moslehi et al. found positive links between specific *XPD* variants in the foetal genome and the risk of placental maldevelopment and PE [27]. Among the Turkish population, Vural et al. studied three SNP sites on DNA repair genes in *XRCC1*, *Apurinic endonuclease 1*, and *XPD*, indicating that these three SNPs may not be related to the onset of PE [28]. Although the study included important genes in the DNA repair system, its small sample size limited its representativeness. In our previous study, we investigated the association between polymorphisms of the other three core genes in the nucleotide excision repair pathway and PE risk and found that *XPA* rs1800975, *XPF* rs1799801, and *XPG* rs17655 were not significantly correlated with the risk of PE as a whole in Chinese Han women [29].

As an initiator of the nucleotide excision repair process, XPC, located in the short arm of chromosome 3 (3p25), plays a key role in DNA damage recognition by binding to the radiation repair 23 B (RAD23B) protein to form the XPC-RAD23B complex. Relevant studies have found that the expression of XPC can downregulate the expression of ROS and lead to a change in metabolism [15]. In addition, many studies have indicated that XPC polymorphisms are associated with the risk of oesophageal squamous cell carcinoma and gastric cardiac adenocarcinoma [30]. Therefore, we hypothesised that the genetic polymorphism of XPC is related to the occurrence of PE to explore the possible pathogenesis of PE.

In our study, 1276 normal pregnant women and 958 patients with PE were enrolled. Genotyping of the two loci rs2228000 and rs2228001 in *XPC*, found that there were significant differences in genotypic and allelic frequencies of rs2228000 between the PE and control groups, and allele T may act as a protect factor. However, no difference was observed in rs2228001 between two groups. To further explore the relationship between them, PE was divided into mild and severe and early-onset and late-onset PE categories. The results generally consistent with those of the large sample data. Then, haplotype analysis showed that AC and AT may be associated with the pathogenesis of PE. Therefore, this study suggests that rs228000 in *XPC* may be associated with the risk of PE in Chinese Han women and allele T was considered as a protective factor in the pathogenesis of PE.

As PE is a complex and polygenetic hereditary disease, determined by both genetic and environmental factors as well as their interactions, the influence of environmental risk factors for PE should not be ignored in our study. Moreover, our results might also be affected by racial and regional differences, suggesting that we should enlarge the scale of subjects and explore more susceptibility genes of PE from multiple regions, to provide the basis for the pathogenesis of PE from the perspective of genetic analysis. Thus, further research is needed to determine the relationship between *XPC* and PE.

## Data Availability

All data and materials are available in this article.

## Abbreviations

PE: Preeclampsia
PCR: Polymerase chain reaction
ROS: Reactive oxygen species
RNS: Reactive nitrogen species
8-OHdG: 8-hydroxy-20-deoxy-guanosin
XPC: Xeroderma pigmentosum complementation group C
NER: Nucleotide excision repair
SNPs: Single nucleotide polymorphisms
RAD23B: Radiation repair 23B
PCOS: Polycystic ovary syndrome
AMH: Anti-Müllerian hormone
XRCC1: X-ray repair cross-complementing group 1
XPD: Xeroderma pigmentosum group D
RAD23B: Radiation repair 23 B
